# Supplementary material for: Genetic diversity and population structure of Phlebotomus argentipes: Vector of Leishmania donovani in Sri Lanka
Source: PLoS One. 2021 Sep 16;16(9):e0256819. doi: 10.1371/journal.pone.0256819 (PMC8445420; doi:10.1371/journal.pone.0256819)
Supplement: S1 Table — Nucleotide identities obtained through NCBI blast results for cox 1 gene. (PDF) [file pone.0256819.s001.pdf]

Table S1- Nucleotide identities obtained through NCBI blast results for cox 1 gene

Descriptior	Scientific N	Max Score	Total Score	Query Cove	E value	Per. ident	Acc. Len	Accession
Phlebotom	Phlebotom	850	850	100%	0	99.78	638	MT585205.
Phlebotom	Phlebotom	850	850	100%	0	99.78	635	MT580112.
Phlebotom	Phlebotom	845	845	100%	0	99.57	630	MT632487.
Phlebotom	Phlebotom	845	845	100%	0	99.57	631	MT632476.
Phlebotom	Phlebotom	845	845	100%	0	99.57	637	MT621222.
Phlebotom	Phlebotom	845	845	100%	0	99.57	637	MT585204.
Phlebotom	Phlebotom	845	845	100%	0	99.57	635	MT580113.
Phlebotom	Phlebotom	845	845	100%	0	99.57	634	MT559510.
Phlebotom	Phlebotom	845	845	100%	0	99.57	620	KT428790.1
Phlebotom	Phlebotom	845	845	100%	0	99.57	644	MN966577
Phlebotom	Phlebotom	845	845	100%	0	99.57	644	MN966576
Phlebotom	Phlebotom	845	845	100%	0	99.57	666	MN966575
Phlebotom	Phlebotom	845	845	100%	0	99.57	669	HQ585371.
Phlebotom	Phlebotom	845	845	100%	0	99.57	673	HQ585369.
Phlebotom	Phlebotom	845	845	100%	0	99.57	671	HQ585368.
Phlebotom	Phlebotom	845	845	100%	0	99.57	673	HQ585367.
Phlebotom	Phlebotom	845	845	100%	0	99.57	672	HQ585366.
Phlebotom	Phlebotom	839	839	100%	0	99.35	642	MT621230.
Phlebotom	Phlebotom	839	839	100%	0	99.35	655	MN966578
Phlebotom	Phlebotom	839	839	100%	0	99.35	671	HQ585373.
Phlebotom	Phlebotom	833	833	98%	0	99.78	573	MT580114.
Phlebotom	Phlebotom	833	833	100%	0	99.14	665	HQ541166.
Phlebotom	Phlebotom	832	832	98%	0	99.56	591	MT577593.
Phlebotom	Phlebotom	828	828	100%	0	98.92	611	KT428792.1
Phlebotom	Phlebotom	828	828	100%	0	98.92	654	JX105038.1
Phlebotom	Phlebotom	813	813	96%	0	99.55	565	KT428789.1
Phlebotom	Phlebotom	804	804	95%	0	99.32	596	KT428791.1
Psychodop	Psychodop	529	529	96%	9.00E-146	88	658	MH281930
Psychodop	Psychodop	529	529	96%	9.00E-146	88	658	MH281892
Lutzomyia	Micropygo	527	527	99%	3.00E-145	87.36	681	GU909480.
Sergentom	Sergentom	527	527	99%	3.00E-145	87.39	663	HQ585389.
Psychodop	Psychodop	523	523	96%	4.00E-144	87.78	658	MH281933
Psychodop	Psychodop	523	523	96%	4.00E-144	87.78	658	MH281932
Psychodop	Psychodop	523	523	96%	4.00E-144	87.78	658	MH281898
Psychodop	Psychodop	523	523	96%	4.00E-144	87.78	658	MH281897
Psychodop	Psychodop	523	523	96%	4.00E-144	87.78	658	MH281895
Psychodop	Psychodop	523	523	96%	4.00E-144	87.78	658	MH281894
Psychodop	Psychodop	523	523	96%	4.00E-144	87.78	658	MH281893
Lutzomyia	Micropygo	521	521	99%	1.00E-143	87.15	681	GU909482.
Sergentom	Sergentom	521	521	99%	1.00E-143	87.17	662	HQ585388.
Psychodop	Psychodop	518	518	96%	2.00E-142	87.56	658	MH281935
Psychodop	Psychodop	518	518	96%	2.00E-142	87.56	658	MH281934
Psychodop	Psychodop	518	518	96%	2.00E-142	87.56	658	MH281931
Psychodop	Psychodop	518	518	96%	2.00E-142	87.56	658	MH281900
Meoneura	Meoneura	516	516	99%	7.00E-142	86.93	588	MG118468
Meoneura	Meoneura	516	516	99%	7.00E-142	86.93	564	MG117103
Meoneura	Meoneura	516	516	99%	7.00E-142	86.93	588	MG119925

Meoneura	Meoneura	516	516	99%	7.00E-142	86.93	588	MF760523.
Meoneura	Meoneura	516	516	99%	7.00E-142	86.93	588	MF760416.
Meoneura	Meoneura	516	516	99%	7.00E-142	86.93	576	MF757988.
Meoneura	Meoneura	516	516	99%	7.00E-142	86.93	588	MF755418.
Meoneura	Meoneura	516	516	99%	7.00E-142	86.93	564	KR462129.1
Phlebotom	Phlebotom	516	516	62%	7.00E-142	98.63	480	JX221068.1
Lutzomyia	Micropygo	516	516	99%	7.00E-142	86.93	681	GU909481.
Lutzomyia	Micropygo	516	516	99%	7.00E-142	86.93	681	GU909479.
Lutzomyia	Micropygo	516	516	99%	7.00E-142	86.93	681	GU909477.
Phlebotom	Phlebotom	514	514	98%	2.00E-141	86.96	655	KU519501.
Phlebotom	Phlebotom	514	514	98%	2.00E-141	86.96	655	KU519500.
Phlebotom	Phlebotom	514	514	98%	2.00E-141	86.96	658	MN812831
Psychodop	Psychodop	512	512	96%	9.00E-141	87.33	658	MH281936
Psychodop	Psychodop	512	512	96%	9.00E-141	87.33	658	MH281899
Psychodop	Psychodop	512	512	96%	9.00E-141	87.33	658	MH281896
Eucalliphor	Eucalliphor	510	510	99%	3.00E-140	86.74	582	MG120245
Eucalliphor	Eucalliphor	510	510	99%	3.00E-140	86.74	582	MG115453
Eucalliphor	Eucalliphor	510	510	99%	3.00E-140	86.74	582	MG112212
Eucalliphor	Eucalliphor	510	510	99%	3.00E-140	86.74	582	MF762748.
Carnidae sp	Carnidae sp	510	510	99%	3.00E-140	86.71	588	MF760043.
Meoneura	Meoneura	510	510	99%	3.00E-140	86.71	588	MF758926.
Eucalliphor	Eucalliphor	510	510	99%	3.00E-140	86.74	600	MF756107.
Eucalliphor	Eucalliphor	510	510	99%	3.00E-140	86.74	546	KR681716.1
Eucalliphor	Eucalliphor	510	510	99%	3.00E-140	86.74	584	KT100917.1
Eucalliphor	Eucalliphor	510	510	99%	3.00E-140	86.74	555	KR679064.1
Carnidae sp	Carnidae sp	510	510	99%	3.00E-140	86.71	579	KR943710.1
Calliphorid	Calliphorid	510	510	99%	3.00E-140	86.74	579	KR498485.1
Meoneura	Meoneura	510	510	99%	3.00E-140	86.71	658	KR439140.1
Eucalliphor	Eucalliphor	510	510	99%	3.00E-140	86.74	623	KM856041.
Eucalliphor	Eucalliphor	510	510	99%	3.00E-140	86.74	588	KM854358.
Eucalliphor	Eucalliphor	510	510	99%	3.00E-140	86.74	555	KM647666.
Eucalliphor	Eucalliphor	510	510	99%	3.00E-140	86.74	636	KM868585.
Eucalliphor	Eucalliphor	510	510	99%	3.00E-140	86.74	635	KM866756.
Eucalliphor	Eucalliphor	510	510	99%	3.00E-140	86.74	631	KM865664.
Eucalliphor	Eucalliphor	510	510	99%	3.00E-140	86.74	639	KM864891.
Eucalliphor	Eucalliphor	510	510	99%	3.00E-140	86.74	613	KM864676.
Eucalliphor	Eucalliphor	510	510	99%	3.00E-140	86.74	564	KM864551.
Meoneura	Meoneura	510	510	99%	3.00E-140	86.71	565	KM862935.
Eucalliphor	Eucalliphor	510	510	99%	3.00E-140	86.74	584	KM571368.
Sergentom	Sergentom	510	510	99%	3.00E-140	86.74	672	HQ585386.
Diptera sp.	Carnidae sp	510	510	99%	3.00E-140	86.71	658	JN291232.1
Carnidae sp	Carnidae sp	510	510	99%	3.00E-140	86.71	658	JF867975.1
Eucalliphor	Eucalliphor	510	510	99%	3.00E-140	86.74	658	HQ945029.
Eucalliphor	Eucalliphor	510	510	99%	3.00E-140	86.74	658	JF868698.1
Phlebotom	Phlebotom	508	508	98%	1.00E-139	86.74	655	KU519499.
Phlebotom	Phlebotom	508	508	98%	1.00E-139	86.74	655	KU519497.
Phlebotom	Phlebotom	508	508	98%	1.00E-139	86.84	658	KR020576.1
Phlebotom	Phlebotom	508	508	98%	1.00E-139	86.81	658	KR020551.1
Phlebotom	Phlebotom	508	508	98%	1.00E-139	86.84	658	KR020549.1
Phlebotom	Phlebotom	508	508	98%	1.00E-139	86.74	658	MT452056.

Phlebotom	Phlebotom	508	508	98%	1.00E-139	86.74	658	MT452055.
Phlebotom	Phlebotom	508	508	98%	1.00E-139	86.74	658	MT452054.
Phlebotom	Phlebotom	508	508	98%	1.00E-139	86.74	658	MT452051.

.1
.1
.1
.1
.1
.1
.1
.1
l
.1
.1
.1
1
1
1
1
1
.1
.1
1
.1
1
.1
l
.
l
l
.1
.1
1
1
.1
.1
.1
.1
.1
.1
.1
1
1
.1
.1
.1
.1
.1
.1
.1

.1
.1
.1
.1
1
.
1
1
1
1
1
.1
.1
.1
.1
.1
.1
.1
.1
.1
.1
.1
1
l
1
1
1
1
.1
.1
.1
.1
.1
.1
.1
.1
.1
.1
.1
1
l

1

1
1
1
1
1
.1

.1
.1
.1
