# Supplementary material for: Genetic diversity and population structure of Phlebotomus argentipes: Vector of Leishmania donovani in Sri Lanka
Source: PLoS One. 2021 Sep 16;16(9):e0256819. doi: 10.1371/journal.pone.0256819 (PMC8445420; doi:10.1371/journal.pone.0256819)
Supplement: S2 Table — Nucleotide identities obtained through NCBI blast results for cytb gene. (PDF) [file pone.0256819.s002.pdf]

Table S2- Nucleotide identities obtained throgh NCBI blast results for cyt b gene

Descriptior	Scientific N	Max Score	Total Score	Query Cove	E value	Per. ident	Acc. Len	Accession
Phlebotom	Phlebotom	364	364	100%	1.00E-96	100	432	KM409508.
Sergentom	Sergentom	215	215	100%	1.00E-51	86.43	430	MK465177.
Sergentom	Sergentom	215	215	100%	1.00E-51	86.36	430	MK465175.
Sergentom	Sergentom	209	209	100%	5.00E-50	85.93	430	MK465176.
Sergentom	Sergentom	209	209	100%	5.00E-50	85.86	502	EF522782.1
Sergentom	Sergentom	204	204	100%	2.00E-48	85.43	502	EF522779.1
Sergentom	Sergentom	204	204	100%	2.00E-48	85.35	502	EF522778.1
Sergentom	Sergentom	198	198	100%	1.00E-46	84.92	421	KP828482.
Sergentom	Sergentom	198	198	100%	1.00E-46	84.85	502	EF522784.1
Sergentom	Sergentom	198	198	100%	1.00E-46	84.85	502	EF522783.1
Sergentom	Sergentom	198	198	100%	1.00E-46	84.85	502	EF522781.1
Sergentom	Sergentom	196	196	91%	4.00E-46	86.19	266	MN853013
Sergentom	Sergentom	193	193	100%	5.00E-45	84.34	430	MK465126.
Phlebotom	Phlebotom	193	193	100%	5.00E-45	84.34	832	KR336650.
Phlebotom	Phlebotom	193	193	100%	5.00E-45	84.34	832	KR336649.
Phlebotom	Phlebotom	193	193	100%	5.00E-45	84.42	828	KR336648.
Phlebotom	Phlebotom	193	193	100%	5.00E-45	84.34	832	KR336647.
Phlebotom	Phlebotom	193	193	100%	5.00E-45	84.42	828	KR336643.
Phlebotom	Phlebotom	193	193	100%	5.00E-45	84.34	832	KR336642.
Sergentom	Sergentom	193	193	100%	5.00E-45	84.42	421	KP828486.
Sergentom	Sergentom	193	193	100%	5.00E-45	84.42	421	KP828485.
Sergentom	Sergentom	193	193	100%	5.00E-45	84.26	266	MN853012
Phlebotom	Phlebotom	193	193	100%	5.00E-45	84.42	720	HQ391907.
Phlebotom	Phlebotom	193	193	100%	5.00E-45	84.42	720	HQ391905.
Phlebotom	Phlebotom	193	193	100%	5.00E-45	84.34	431	HM803196
Phlebotom	Phlebotom	193	193	100%	5.00E-45	84.42	714	AF161211.
Sergentom	Sergentom	187	187	100%	2.00E-43	83.84	430	MK465158.
Sergentom	Sergentom	187	187	100%	2.00E-43	83.84	430	MK465135.
Thitarodes	Thitarodes	187	187	100%	2.00E-43	83.92	15290	KU053201.
Phlebotom	Phlebotom	187	187	100%	2.00E-43	83.84	832	KR336651.
Sergentom	Sergentom	187	187	100%	2.00E-43	83.76	266	MN853018
Phlebotom	Phlebotom	187	187	100%	2.00E-43	83.92	313	HQ391913.
Phlebotom	Phlebotom	187	187	100%	2.00E-43	83.92	313	HQ391912.
Phlebotom	Phlebotom	187	187	100%	2.00E-43	83.92	313	HQ391911.
Phlebotom	Phlebotom	187	187	100%	2.00E-43	83.84	431	HM803195
Phlebotom	Phlebotom	187	187	100%	2.00E-43	83.92	714	HQ023285.
Phlebotom	Phlebotom	187	187	100%	2.00E-43	83.92	714	HQ023284.
Phlebotom	Phlebotom	187	187	100%	2.00E-43	83.92	297	GQ169349.
Phlebotom	Phlebotom	187	187	100%	2.00E-43	83.92	312	GQ169345.
Phlebotom	Phlebotom	187	187	100%	2.00E-43	83.92	303	GQ169344.
Phlebotom	Phlebotom	187	187	100%	2.00E-43	83.92	714	AF161212.
Phlebotom	Phlebotom	187	187	100%	2.00E-43	83.92	714	AF161210.
Phlebotom	Phlebotom	187	187	100%	2.00E-43	83.92	441	U94473.1
Sergentom	Sergentom	182	182	100%	1.00E-41	83.42	412	KY451833.
Sergentom	Sergentom	182	182	100%	1.00E-41	83.33	409	KY451819.
Sergentom	Sergentom	182	182	100%	1.00E-41	83.42	409	KY451817.
Phlebotom	Phlebotom	182	182	100%	1.00E-41	83.33	832	KR336659.

Phlebotom	Phlebotom	182	182	100%	1.00E-41	83.33	832	KR336658.1
Phlebotom	Phlebotom	182	182	100%	1.00E-41	83.33	832	KR336657.1
Sergentom	Sergentom	182	182	100%	1.00E-41	83.5	421	KP828508.1
Sergentom	Sergentom	182	182	100%	1.00E-41	83.42	421	KP828503.1
Phlebotom	Phlebotom	182	182	98%	1.00E-41	83.67	652	MT452059.
Phlebotom	Phlebotom	182	182	98%	1.00E-41	83.67	652	MT452058.
Phlebotom	Phlebotom	182	182	98%	1.00E-41	83.67	652	MT452057.
Phlebotom	Phlebotom	182	182	98%	1.00E-41	83.67	652	MT452053.
Grassomyia	Grassomyia	182	182	100%	1.00E-41	83.5	266	MN853033
Phlebotom	Phlebotom	182	182	98%	1.00E-41	83.67	652	MN812836
Sergentom	Sergentom	182	182	100%	1.00E-41	83.5	901	JF766980.1
Phlebotom	Phlebotom	182	182	100%	1.00E-41	83.42	313	HQ391910.
Sergentom	Sergentom	182	182	100%	1.00E-41	83.42	429	HM803198
Phlebotom	Phlebotom	182	182	100%	1.00E-41	83.42	714	HQ023283.
Phlebotom	Phlebotom	182	182	100%	1.00E-41	83.42	714	AF161209.1
Sergentom	Sergentom	182	182	100%	1.00E-41	83.5	282	U94478.1
Diestramin	Diestramin	176	176	100%	5.00E-40	82.91	16060	KX057740.1
Sergentom	Sergentom	176	176	100%	5.00E-40	83	421	KP828506.1
Sergentom	Sergentom	176	176	100%	5.00E-40	82.91	421	KP828505.1
Sergentom	Sergentom	176	176	100%	5.00E-40	83	421	KP828489.1
Sergentom	Sergentom	176	176	100%	5.00E-40	83	421	KP828488.1
Sergentom	Sergentom	176	176	100%	5.00E-40	83	421	KP828487.1
Phlebotom	Phlebotom	176	176	98%	5.00E-40	83.16	652	MT452052.
Sergentom	Sergentom	176	176	100%	5.00E-40	83	901	JF766978.1
Sergentom	Sergentom	176	176	100%	5.00E-40	83	901	JF766977.1
Sergentom	Sergentom	176	176	100%	5.00E-40	83	901	JF766976.1
Sergentom	Sergentom	176	176	100%	5.00E-40	83	270	HM803209
Sergentom	Sergentom	176	176	100%	5.00E-40	83	440	HM803205
Thitarodes	Thitarodes	171	171	100%	2.00E-38	82.32	15362	NC_04477C
Phlebotom	Phlebotom	171	171	100%	2.00E-38	82.23	662	KX024721.1
Sergentom	Sergentom	171	171	100%	2.00E-38	82.41	469	KP702250.1
Sergentom	Sergentom	171	171	100%	2.00E-38	82.5	421	KP828504.1
Sergentom	Sergentom	171	171	100%	2.00E-38	82.41	421	KP828484.1
Sergentom	Sergentom	171	171	100%	2.00E-38	82.41	421	KP828457.1
Phlebotom	Phlebotom	171	171	100%	2.00E-38	82.41	437	KF680832.1
Phlebotom	Phlebotom	171	171	100%	2.00E-38	82.41	437	KF680828.1
Grassomyia	Grassomyia	171	171	100%	2.00E-38	82.5	266	MN853032
Phlebotom	Phlebotom	171	171	100%	2.00E-38	82.41	312	GQ169338.
Phlebotom	Phlebotom	165	165	100%	1.00E-36	81.91	312	MK991773.
Diestramin	Diestramin	165	165	100%	1.00E-36	82.09	16346	KX057718.1
Sergentom	Sergentom	165	165	100%	1.00E-36	81.91	469	KP702264.1
Sergentom	Sergentom	165	165	100%	1.00E-36	81.91	469	KP702262.1
Sergentom	Sergentom	165	165	100%	1.00E-36	81.91	469	KP702260.1
Sergentom	Sergentom	165	165	100%	1.00E-36	81.91	469	KP702257.1
Sergentom	Sergentom	165	165	100%	1.00E-36	81.91	469	KP702256.1
Sergentom	Sergentom	165	165	100%	1.00E-36	81.91	421	KP828501.1
Sergentom	Sergentom	165	165	100%	1.00E-36	81.91	421	KP828497.1
Sergentom	Sergentom	165	165	100%	1.00E-36	81.91	421	KP828495.1
Sergentom	Sergentom	165	165	100%	1.00E-36	81.91	421	KP828494.1
Sergentom	Sergentom	165	165	100%	1.00E-36	81.91	421	KP828493.1

Sergentom	Sergentom	165	165	100%	1.00E-36	81.91	421	KP828478.1
Sergentom	Sergentom	165	165	100%	1.00E-36	81.91	421	KP828477.1
Conosia irr	Conosia irr	165	165	100%	1.00E-36	81.82	14634	MK864103.

□

.1
.1
.1
.1
l
l
l
1
l
l
l
.1
.1
1
1
1
1
1
1
1
1
.1
1
1
.1
1
.1
.1
1
1
.1
1
1
1
.1
1
1
1
1
1
1
1

1
1
1
1

1
1
1
1
.1
.1
.1
.1
.1
.1

1
.1
1
1

1
1
1
1
1
1
.1

.1
.1
).1
1
1
1
1
1
1
1
.1
1
.1
1
1
1
1
1
1
1
1
1
1
1

1
1
.1
