# Supplementary material for: Genetic diversity and population structure of Phlebotomus argentipes: Vector of Leishmania donovani in Sri Lanka
Source: PLoS One. 2021 Sep 16;16(9):e0256819. doi: 10.1371/journal.pone.0256819 (PMC8445420; doi:10.1371/journal.pone.0256819)
Supplement: S3 Table — Nucleotide identities obtained through NCBI blast results for ITS2 region. (PDF) [file pone.0256819.s003.pdf]

Table S3- Nucleotide identities obtained throgh NCBI blast results for ITS2 region

Descriptior	Scientific N	Max Score	Total Score	Query Cove	E value	Per. ident	Acc. Len	Accession
Phlebotom	Phlebotom	285	285	61%	2.00E-72	89.57	246	KF416360.1
Phlebotom	Phlebotom	283	283	50%	6.00E-72	94.15	246	KF416361.1
Phlebotom	Phlebotom	283	283	50%	6.00E-72	94.15	246	KF416359.1
Phlebotom	Phlebotom	233	233	61%	6.00E-57	86.09	238	KF416354.1
Phlebotom	Phlebotom	228	228	61%	3.00E-55	85.65	238	KF416357.1
Phlebotom	Phlebotom	182	182	50%	2.00E-41	84.9	408	KM409470.
Phlebotom	Phlebotom	174	174	50%	4.00E-39	85.03	382	KM409469.
Phlebotom	Phlebotom	134	134	29%	6.00E-27	89.29	408	KC412877.1
Phlebotom	Phlebotom	130	130	66%	8.00E-26	77.73	401	KX527898.1
Phlebotom	Phlebotom	128	128	66%	3.00E-25	77.65	384	KX527897.1
Phlebotom	Phlebotom	128	128	66%	3.00E-25	77.52	382	KM409461.
Phlebotom	Phlebotom	126	126	22%	1.00E-24	94.05	384	KM409460.
Phlebotom	Phlebotom	126	126	22%	1.00E-24	94.05	385	KM409459.
Phlebotom	Phlebotom	124	124	29%	4.00E-24	87.5	409	KC412878.1
Phlebotom	Phlebotom	117	117	20%	6.00E-22	94.74	445	KM409458.
Phlebotom	Phlebotom	117	117	20%	6.00E-22	94.74	448	KM409457.
Phlebotom	Phlebotom	117	117	20%	6.00E-22	94.74	444	KM409456.
Phlebotom	Phlebotom	117	117	20%	6.00E-22	94.74	446	KM409455.
Phlebotom	Phlebotom	117	117	20%	6.00E-22	94.74	445	KM409454.
Phlebotom	Phlebotom	117	117	20%	6.00E-22	94.74	445	KM409453.
Phlebotom	Phlebotom	106	106	30%	1.00E-18	84.82	427	KC412906.1
Phlebotom	Phlebotom	106	106	30%	1.00E-18	84.82	411	KC412903.1
Phlebotom	Phlebotom	106	106	30%	1.00E-18	84.82	422	KC412902.1
Phlebotom	Phlebotom	106	106	30%	1.00E-18	84.82	414	JF729350.1
Phlebotom	Phlebotom	106	106	30%	1.00E-18	84.82	402	AF205527.1
Phlebotom	Phlebotom	102	102	29%	2.00E-17	84.82	369	KM409471.
Phlebotom	Phlebotom	102	102	29%	2.00E-17	84.82	415	KC412911.1
Phlebotom	Phlebotom	102	102	29%	2.00E-17	84.82	417	KC412875.1
Phlebotom	Phlebotom	102	102	29%	2.00E-17	84.55	294	HM747280
Phlebotom	Phlebotom	102	102	29%	2.00E-17	84.55	294	HM747279
Phlebotom	Phlebotom	102	102	29%	2.00E-17	84.55	458	GU385746.
Phlebotom	Phlebotom	100	100	30%	6.00E-17	83.33	421	KY564192.1
Phlebotom	Phlebotom	100	100	30%	6.00E-17	83.33	415	KC412907.1
Phlebotom	Phlebotom	100	100	30%	6.00E-17	83.33	430	KC412905.1
Phlebotom	Phlebotom	99	99	30%	2.00E-16	83.19	369	KM409472.
Phlebotom	Phlebotom	99	99	30%	2.00E-16	83.19	418	JF729351.1
Phlebotom	Phlebotom	97.1	97.1	30%	8.00E-16	82.76	433	KY564196.1
Phlebotom	Phlebotom	97.1	97.1	29%	8.00E-16	83.04	415	KC412904.1
Phlebotom	Phlebotom	97.1	97.1	22%	8.00E-16	88.24	413	KC412894.1
Phlebotom	Phlebotom	97.1	97.1	22%	8.00E-16	88.24	413	KC412893.1
Phlebotom	Phlebotom	97.1	97.1	22%	8.00E-16	88.24	413	KC412892.1
Phlebotom	Phlebotom	97.1	97.1	25%	8.00E-16	86.46	440	KF186427.1
Phlebotom	Phlebotom	97.1	97.1	34%	8.00E-16	81.2	505	JN230424.1
Phlebotom	Phlebotom	97.1	97.1	29%	8.00E-16	83.64	436	AF205525.1
Phlebotom	Phlebotom	95.3	95.3	20%	3.00E-15	89.47	441	MN341184
Phlebotom	Phlebotom	95.3	95.3	20%	3.00E-15	89.47	441	MN341182
Phlebotom	Phlebotom	95.3	95.3	30%	3.00E-15	82.76	422	KY564194.1

Phlebotom	Phlebotom	95.3	95.3	20%	3.00E-15	89.47	365	KM409464.
Phlebotom	Phlebotom	95.3	95.3	20%	3.00E-15	89.47	367	KM409463.
Phlebotom	Phlebotom	95.3	95.3	20%	3.00E-15	89.47	366	KM409462.
Phlebotom	Phlebotom	95.3	95.3	20%	3.00E-15	89.47	459	AY700676.
Phlebotom	Phlebotom	95.3	95.3	20%	3.00E-15	89.47	461	AY425624.
Phlebotom	Phlebotom	95.3	95.3	21%	3.00E-15	88.89	390	AF205526.
Phlebotom	Phlebotom	93.5	93.5	13%	1.00E-14	100	428	KY564197.
Phlebotom	Phlebotom	93.5	93.5	22%	1.00E-14	87.06	434	KY564190.
Phlebotom	Phlebotom	93.5	93.5	28%	1.00E-14	83.96	369	KR020607.
Phlebotom	Phlebotom	93.5	93.5	28%	1.00E-14	83.96	411	KR020594.
Phlebotom	Phlebotom	93.5	93.5	13%	1.00E-14	100	403	KC412909.
Phlebotom	Phlebotom	93.5	93.5	13%	1.00E-14	100	399	KC412908.
Phlebotom	Phlebotom	93.5	93.5	20%	1.00E-14	89.47	458	KC412876.
Sergentom	Sergentom	93.5	93.5	34%	1.00E-14	81.06	452	JQ255479.1
Phlebotom	Phlebotom	93.5	93.5	28%	1.00E-14	83.96	489	JN172919.1
Phlebotom	Phlebotom	93.5	93.5	28%	1.00E-14	83.96	422	GU593685.
Phlebotom	Phlebotom	93.5	93.5	34%	1.00E-14	81.06	388	GU593683.
Phlebotom	Phlebotom	93.5	93.5	21%	1.00E-14	88.75	450	GU385754.
Phlebotom	Phlebotom	93.5	93.5	28%	1.00E-14	83.96	422	EF408782.1
Phlebotom	Phlebotom	93.5	93.5	34%	1.00E-14	81.06	424	EF408781.1
Phlebotom	Phlebotom	91.6	91.6	29%	4.00E-14	83.04	458	GU385752.
Phlebotom	Phlebotom	91.6	91.6	28%	4.00E-14	83.18	426	EF408780.1
Phlebotom	Phlebotom	89.8	89.8	21%	1.00E-13	87.5	285	HM747286
Phlebotom	Phlebotom	89.8	89.8	21%	1.00E-13	87.5	285	HM747283
Phlebotom	Phlebotom	89.8	89.8	29%	1.00E-13	83.04	458	GU385751.
Phlebotom	Phlebotom	89.8	89.8	21%	1.00E-13	87.5	446	GU385749.
Phlebotom	Phlebotom	89.8	89.8	30%	1.00E-13	82.3	400	AF205528.
Phlebotom	Phlebotom	87.9	87.9	28%	5.00E-13	83.02	486	KX527895.
Phlebotom	Phlebotom	87.9	87.9	26%	5.00E-13	83.84	297	KF680846.1
Phlebotom	Phlebotom	87.9	87.9	13%	5.00E-13	98	285	HM747285
Phlebotom	Phlebotom	87.9	87.9	13%	5.00E-13	98	285	HM747284
Phlebotom	Phlebotom	87.9	87.9	13%	5.00E-13	98	290	HM747278
Phlebotom	Phlebotom	87.9	87.9	13%	5.00E-13	98	290	HM747277
Phlebotom	Phlebotom	87.9	87.9	13%	5.00E-13	98	290	HM747276
Phlebotom	Phlebotom	87.9	87.9	13%	5.00E-13	98	458	GU385750.
Phlebotom	Phlebotom	87.9	87.9	13%	5.00E-13	98	459	GU385747.
Phlebotom	Phlebotom	87.9	87.9	13%	5.00E-13	98	395	AF205523.
Phlebotom	Phlebotom	84.2	84.2	35%	6.00E-12	79.7	415	KX527914.
Phlebotom	Phlebotom	84.2	84.2	35%	6.00E-12	79.7	400	KX527908.
Phlebotom	Phlebotom	84.2	84.2	35%	6.00E-12	79.7	403	KX527907.
Phlebotom	Phlebotom	84.2	84.2	35%	6.00E-12	79.7	462	KX527906.
Phlebotom	Phlebotom	84.2	84.2	35%	6.00E-12	79.7	423	KX527902.
Phlebotom	Phlebotom	84.2	84.2	35%	6.00E-12	79.7	420	KX527901.
Sergentom	Sergentom	84.2	84.2	32%	6.00E-12	80	484	KX527900.
Phlebotom	Phlebotom	84.2	84.2	35%	6.00E-12	79.7	454	KP773297.
Phlebotom	Phlebotom	84.2	84.2	34%	6.00E-12	80	455	KP773296.
Phlebotom	Phlebotom	84.2	84.2	35%	6.00E-12	79.7	454	KP773295.
Phlebotom	Phlebotom	84.2	84.2	35%	6.00E-12	79.7	455	KP756625.
Phlebotom	Phlebotom	84.2	84.2	35%	6.00E-12	79.7	366	KR020606.
Phlebotom	Phlebotom	84.2	84.2	35%	6.00E-12	79.7	392	KR020605.

Phlebotom	Phlebotom	84.2	84.2	35%	6.00E-12	79.7	296	KR020604.[illegible]
Phlebotom	Phlebotom	84.2	84.2	35%	6.00E-12	79.7	400	KR020602.[illegible]
Phlebotom	Phlebotom	84.2	84.2	35%	6.00E-12	79.7	285	KR020601.[illegible]

l
l
l
l
l
.1
.1
1
1
1
.1
.1
.1
1
.1
.1
.1
.1
.1
.1
1
1
1

l
.1
1
1
.1
.1
1
l
1
1
.1

l
1
1
1
1
l
l
1
.1
.1
l

.1
.1
.1
1
1
1
1
1
1
1
1
1
1
1
1
1
1
1
1
1
1
1
.1
.1
1
1
1
1
1
.1
.1
.1
.1
.1
1
1
1
1
1
1
1
1
1
1
1
1
1
1
1
1

1
1
1
