# Supplementary material for: Genetic diversity and population structure of Phlebotomus argentipes: Vector of Leishmania donovani in Sri Lanka
Source: PLoS One. 2021 Sep 16;16(9):e0256819. doi: 10.1371/journal.pone.0256819 (PMC8445420; doi:10.1371/journal.pone.0256819)
Supplement: S4 Table — Analysis of Molecular Variance (AMOVA) of cox 1, cytb genes and ITS2 region using Arlequin software (version 3.11) (cmpg.unibe.ch/software/arlequin3/). (PDF) [file pone.0256819.s004.pdf]

Table S4 – Analysis of Molecular Variance (AMOVA) of *cox 1*, *cytb* genes and ITS2 region.

Project information:

NbSamples = 42

DataType = DNA

GenotypicData = 0

Analysis of Molecular Variance:

No. of Permutations = 1000

Population pairwise Fst values:

No. of permutations for significance = 100

No. of permutations for Mantel test = 1000

Distance matrix:

Compute distance matrix

Molecular distance : Pairwise difference

Gamma a value = 0

==

AMOVA ANALYSIS

==

Genetic structure to test :

No. of Groups = 1

[[Structure]]

StructureName = "New Edited Structure"

NbGroups = 1

IndividualLevel = 0

DistMatLabel = ""

Group={

"Mirigama"

"Mamadala"

"Thalawa"

"Pannala"

}

Distance method: Pairwise difference

AMOVA design and results : **For *cox 1* gene**

Reference: Weir, B.S. and Cockerham, C.C. 1984.

Excoffier, L., Smouse, P., and Quattro, J. 1992.

Weir, B. S., 1996.

Source of variation	d.f.	Sum of squares	Variance components	Percentage of variation
Among populations	3	302.804	3.76909 Va	5.78
Within populations	38	2332.791	61.38923 Vb	94.22
Total	41	2635.595	65.15833	

Fixation Index FST : 0.05785

Significance tests (1023 permutations)

Va and FST : P(rand. value > obs. value) = 0.19648

P(rand. value = obs. value) = 0.00000

P-value = 0.19648+-0.01208

===

== Comparisons of pairs of population samples

===

List of labels for population samples used below:

Label Population name

----- ---------------

1: Mirigama

2: Mamadala

3: Thalawa

4: Pannala

Population pairwise FSTs

Distance method: Pairwise difference

	1	2	3	4
1	0.00000			
2	-0.08011	0.00000		
3	-0.09859	-0.09670	0.00000	
4	0.24943	0.13873	0.19531	0.00000

FST P values

Number of permutations : 110

	1	2	3	4
1	*			
2	0.56757+-0.0430	*		
3	0.85586+-0.0390	0.95495+-0.0203	*	
4	0.09910+-0.0286	0.16216+-0.0466	0.08108+-0.0252	*

Matrix of significant Fst P values

Significance Level=0.0500

AMOVA design and results : **For *cytb* gene**

Reference: Weir, B.S. and Cockerham, C.C. 1984.

Excoffier, L., Smouse, P., and Quattro, J. 1992.

Weir, B. S., 1996.

Source of variation	d.f.	Sum of squares	Variance components	Percentage of variation
Among populations	3	250.681	7.04490 Va	42.21
Within populations	38	366.509	9.64498 Vb	57.79
Total	41	617.190	16.68987	

Fixation Index FST : 0.42211

Significance tests (1023 permutations)

Va and FST : P(rand. value > obs. value) = 0.00196

P(rand. value = obs. value) = 0.00000

P-value = 0.00196+-0.00136

== Comparisons of pairs of population samples

===

List of labels for population samples used below:

Label	Population name
-----	---------------
1:	Mirigama
2:	Mamadala
3:	Thalawa
4:	Pannala

Population pairwise FSTs

Distance method: Pairwise difference

	1	2	3	4
1	0.00000			
2	-0.03186	0.00000		
3	-0.00144	0.00711	0.00000	
4	0.53985	0.55809	0.35835	0.00000

FST P values

Number of permutations : 110

	1	2	3	4
1	*			
2	0.61261+-0.0588	*		
3	0.48649+-0.0511	0.33333+-0.0430	*	
4	0.01802+-0.0121	0.00000+-0.0000	0.09009+-0.0303	*

Matrix of significant Fst P values

Significance Level=0.0500

AMOVA design and results : **For ITS2 region**

Reference: Weir, B.S. and Cockerham, C.C. 1984.

Excoffier, L., Smouse, P., and Quattro, J. 1992.

Weir, B. S., 1996.

Source of variation	d.f.	Sum of squares	Variance components	Percentage of variation
Among populations	3	14.726	-0.70232 Va	-6.07
Within populations	38	466.536	12.27727 Vb	106.07
Total	41	481.262	11.57496	

Fixation Index FST : -0.06068

Significance tests (1023 permutations)

Va and FST : P(rand. value > obs. value) = 0.75171

P(rand. value = obs. value) = 0.02835

P-value = 0.78006+-0.01426

===

== Comparisons of pairs of population samples

===

List of labels for population samples used below:

Label	Population name
-----	---------------
1:	Mirigama
2:	Mamadala
3:	Thalawa
4:	Pannala

Population pairwise FSTs

Distance method: Pairwise difference

	1	2	3	4
1	0.00000			
2	0.01579	0.00000		
3	-0.08035	-0.05162	0.00000	
4	-0.08952	-0.05307	-0.10333	0.00000

FST P values

Number of permutations : 110

	1	2	3	4
1	*			
2	0.63063+-0.0272	*		
3	0.83784+-0.0274	0.58559+-0.0280	*	
4	0.99099+-0.0030	0.53153+-0.0394	0.99099+-0.0030	*

Matrix of significant Fst P values

Significance Level=0.0500
