# Supplementary material for: Genetic diversity and population structure of Phlebotomus argentipes: Vector of Leishmania donovani in Sri Lanka
Source: PLoS One. 2021 Sep 16;16(9):e0256819. doi: 10.1371/journal.pone.0256819 (PMC8445420; doi:10.1371/journal.pone.0256819)
Supplement: Attachment — Submitted filename: Response to Reviewers.docx [file pone.0256819.s005.docx]

***Author responses to editor’s and reviewers comments:***

*Additional Editor’s Comments:*

*Q: it is pivotal to modify the ms. according to comments of both reviewers and focusing the population genetic analysis on microsatellite loci; otherwise, you have to limit the study only on the molecular typing of sand fly species examined.*

R: The current study was designed to determine the population genetic structure of sand flies using two mitochondrial markers *cox 1*, *cytb* and a ribosomal region ITS2. However, no microsatellite loci were used for genotyping due their unavailability of such markers in *P. argentipes*. While we as a group, continue our efforts in developing such robust markers for *P. argentipes*, the authors believe the selected antigenic loci of *P. argentipes* are good enough for the said purpose due to the following reasons:

Previous studies have successfully used *cox 1*and *cyb* as the genetic marker in analysing the population genetic structure of insects including sand flies.

Ebrahimi, *et al.* Genetic dynamics in the sand fly (Diptera: Psychodidae) nuclear and mitochondrial genotypes: evidence for vector adaptation at the border of Iran with Iraq. *Parasites Vectors* **9,** 319 (2016). <https://doi.org/10.1186/s13071-016-1603-5>.

Flanley, *et al.* Population genetics analysis of *Phlebotomus papatasi* sand flies from Egypt and Jordan based on mitochondrial cytochrome b haplotypes. *Parasites Vectors.* 2018; 11, 214, doi:10.1186/s13071-018-2785-9.

Guernaoui, *et al.* Population Genetics of *Phlebotomus papatasi* from Endemic and Nonendemic Areas for Zoonotic Cutaneous Leishmaniasis in Morocco, as Revealed by Cytochrome Oxidase Gene Subunit I Sequencing. *Microorganisms*. 2020; *8*(7): 1010.

Arrivillaga *et al.* Phylogeography of the neotropical sand fly *Lutzomyia longipalpis* inferred from mitochondrial DNA sequences. Infect Genet Evol. 2002; 2: 83-95.

Latrofaa *et al.* Multilocus molecular and phylogenetic analysis of phlebotomine sand flies ( Diptera : Psychodidae ) from southern Italy. Acta trop. 2011;119:91–8. doi:10.1016/j.actatropica.2011.04.013.

Cohnstaedt *et al*. Phylogenetics of the phlebotomine sand fly group *Verrucarum* (Diptera: Psychodidae: Lutzomyia). Am J Trop Med Hyg. 2011;84(6):913–22.

Weeraratne *et al.* Genetic diversity and population structure of malaria vector mosquitoes *Anopheles subpictus, Anopheles peditaeniatus,* and *Anopheles vagus* in five districts of Sri Lanka. Malar. J. 2018; 17: 271. <https://doi.org/10.1186/s12936-018-2419-x>.

Gene flow also successfully determined using these markers in following studies.

Marcondes *et al*. Introgression between *Lutzomyia intermedia* and both *Lu. neivai* and *Lu. whitmani*, and their roles as vectors of *Leishmania braziliensis*. Trans R Soc Trop Med Hyg. 1997; 91(6): 725-6.

[Belen](https://onlinelibrary.wiley.com/action/doSearch?ContribAuthorStored=Belen%2C+Asli) *et al.* Genetic structures of sand fly (Diptera: Psychodidae) populations in a leishmaniasis endemic region of Turkey. Journal of vector biology. 2011; 36 (Supplement 1): 32-48. <https://doi.org/10.1111/j.1948-7134.2011.00110.x>.

Studies appear below have proven that ITS regions could successfully determine the population genetic structure of many organisms including parasites and mosquitoes. Further, Manni et al (2015) have used microsatellites and ITS2 as the genetic markers and have proven that both markers are equally successful in determining the population genetic structure.

Manni, M., Gomulski, L.M., Aketarawong, N. *et al.* Molecular markers for analyses of intraspecific genetic diversity in the Asian Tiger mosquito, *Aedes albopictus* . *Parasites Vectors* **8,** 188 (2015). <https://doi.org/10.1186/s13071-015-0794-5>

Villalobos, G., Orozco-Mosqueda, G.E., Lopez-Perez, M. *et al.* Suitability of internal transcribed spacers (ITS) as markers for the population genetic structure of *Blastocystis* spp. *Parasites Vectors* **7,** 461 (2014). <https://doi.org/10.1186/s13071-014-0461-2>.

Latrofaa MS, Dantas-Torresa F, Weigla S, Taralloa VD, Parisib A, Traversac D, et al. Multilocus molecular and phylogenetic analysis of phlebotomine sand flies ( Diptera : Psychodidae ) from southern Italy. Acta trop. 2011;119:91–8. doi:10.1016/j.actatropica.2011.04.013.

*Journal Requirements:*

*1. Please ensure that your manuscript meets PLOS ONE's style requirements, including those for file naming. The PLOS ONE style templates can be found at*

[*https://journals.plos.org/plosone/s/file?id=wjVg/PLOSOne_formatting_sample_main_body.pdf*](https://journals.plos.org/plosone/s/file?id=wjVg/PLOSOne_formatting_sample_main_body.pdf) *and*

*https://journals.plos.org/plosone/s/file?id=ba62/PLOSOne_formatting_sample_title_authors_affiliations.pdf*

The manuscript was checked and changed according to the guidelines provided in the PLOS ONE journal and further both above links also were used.

*2. We note that Figure 1 in your submission contain map images which may be copyrighted. PLOS content is published under the Creative Commons Attribution License (CC BY 4.0), which means that the manuscript, images, and Supporting Information files will be freely available online, and any third party is permitted to access, download, copy, distribute, and use these materials in any way, even commercially, with proper attribution. For these reasons, we cannot publish previously copyrighted maps or satellite images created using proprietary data, such as Google software (Google Maps, Street View, and Earth). For more information, see our copyright guidelines:* [*http://journals.plos.org/plosone/s/licenses-and-copyright*](http://journals.plos.org/plosone/s/licenses-and-copyright)*.*

*We require you to either (1) present written permission from the copyright holder to publish these figures specifically under the CC BY 4.0 license, or (2) remove the figures from your submission:*

*2.1.    You may seek permission from the original copyright holder of Figure 1 to publish the content specifically under the CC BY 4.0 license.*

*We recommend that you contact the original copyright holder with the Content Permission Form (*[*http://journals.plos.org/plosone/s/file?id=7c09/content-permission-form.pdf*](http://journals.plos.org/plosone/s/file?id=7c09/content-permission-form.pdf)*) and the following text:*

*“I request permission for the open-access journal PLOS ONE to publish XXX under the Creative Commons Attribution License (CCAL) CC BY 4.0 (*[*http://creativecommons.org/licenses/by/4.0/*](http://creativecommons.org/licenses/by/4.0/)*). Please be aware that this license allows unrestricted use and distribution, even commercially, by third parties. Please reply and provide explicit written permission to publish XXX under a CC BY license and complete the attached form.”*

*Please upload the completed Content Permission Form or other proof of granted permissions as an "Other" file with your submission.*

*In the figure caption of the copyrighted figure, please include the following text: “Reprinted from [ref] under a CC BY license, with permission from [name of publisher], original copyright [original copyright year].*

Agreed your kind request and done it. The figure 1 was reprinted from authors’ previous work [Pathirage *et al.* 2020]. Thus, the written permission from the copyright holder to publish these figures specifically under the CC BY 4.0 license was obtained and upload the completed Content Permission Form and the original figure in the previous work as an “Other” file when submitting the manuscript. Further, in the figure caption “Reprinted from authors’ previous work [Pathirage *et al.* 2020]” was stated in line 105-106 (page number 5) under Materials and Method section in the manuscript.

Reviewer Comments:

*Reviewer 1:*

*Q1: Is the manuscript technically sound, and do the data support the conclusions?

The manuscript must describe a technically sound piece of scientific research with data that supports the conclusions. Experiments must have been conducted rigorously, with appropriate controls, replication, and sample sizes. The conclusions must be drawn appropriately based on the data presented.*

*Reviewer #1: No*

*Reviewer #2: Partly*

R1: A minimum of 10 sequences of each marker from each study site was used in the analysis. Previous published work on population genetic structure analysis have used sample numbers between 6-10 from a single study location and have obtained successful results. I suggest adding one statement to the discussion in line 342-343 and changing the following sentence in the conclusion in line 370-373, 375-377–

‘Gene flow was evident with lack of population differentiation even between geographically distant populations of *P. argentipes* perhaps due to absence of geographic barriers and the continuity of habitats.’

‘Results also validated the use of *cox 1, cytb* genes and ITS2 region as a tool in understanding the population genetic structure of Phlebotominae sand flies in Sri Lanka.’

As,

Both *cox 1* and ITS2 indicated a gene flow with lack of population differentiation even between geographically distant populations of *P. argentipes* perhaps due to absence of geographic barriers and the continuity of habitats.

Results showed the possible use of *cox 1, cytb* genes and ITS2 region as markers in determining the population genetic structure of Phlebotominae sand flies in Sri Lanka.

*Q2. Has the statistical analysis been performed appropriately and rigorously?*

*Reviewer #1: No*

*Reviewer #2: Yes*

 R2: Standard statistical software and analysis were used in the study by referring to previous published work.

*Q3: The markers used in this paper are not suitable for assessing population genetic structure and gene flow among populations. At this aim, microsatellite or SNPs markers should be used. Consequently the inferred conclusions are not supported by genetic data.
The genetic analyses carried out in this paper revealed unique sequences of “all genomic regions studied except the cox 1 gene in 21 flies that aligned with those from Kerala, India and cytb gene of 4 flies that aligned with those isolated earlier from Sri Lanka and 3 from Madagascar”.
The authors say that Cox 1 gene and ITS 2 region analyses revealed gene flow between the study sites”. However, the lack of differentiation among populations is due to incomplete lineage sorting and the lack of power of these markers for intra-specific studies.*

R3: The current study was designed to determine the population genetic structure of sand flies using two mitochondrial markers *cox 1*, *cytb* and a ribosomal region ITS2. However, no microsatellite loci were used for genotyping due their unavailability of such markers in *P. argentipes*. While we as a group, continue our efforts in developing such robust markers for *P. argentipes*, the authors believe the selected antigenic loci of *P. argentipes* are good enough for the said purpose due to the following reasons:

Most of the studies have been conducted to investigate population genetic analysis using these markers (Ebrahimi *et al.* 2016, Flanley *et al,* 2018, Guernaoui *et al.* 2020, Arrivillaga *et al.* 2002, Cohnstaedt *et al*. 2011, Testa *et al.* 2002, Latrofaa *et al.* 2011, Weeraratne *et al.* 2018).

Gene flow also successfully determined using these markers in following studies.

(Marcondes *et al.* 1997, Belen *et al. 2*011).

Further, several studies have conducted using one or two of the markers used in the present study and microsattelites and have proved that both could be used sucessfully in population genetic structure analysis (Manni *et al.* 2015, Villalobos *et al.* 2014).

we suggest changing the following sentence in the conclusion in line 370-373, 375-377–

‘Gene flow was evident with lack of population differentiation even between geographically distant populations of *P. argentipes* perhaps due to absence of geographic barriers and the continuity of habitats.’

‘Results also validated the use of *cox 1, cytb* genes and ITS2 region as a tool in understanding the population genetic structure of Phlebotominae sand flies in Sri Lanka.’

As,

Both *cox 1* and ITS2 indicated a gene flow with lack of population differentiation even between geographically distant populations of *P. argentipes* perhaps due to absence of geographic barriers and the continuity of habitats.

Results showed the possible use of *cox 1, cytb* genes and ITS2 region as markers in determining the population genetic structure of Phlebotominae sand flies in Sri Lanka.

*Reviewer 2:*

*Q1: Is the manuscript technically sound, and do the data support the conclusions?

The manuscript must describe a technically sound piece of scientific research with data that supports the conclusions. Experiments must have been conducted rigorously, with appropriate controls, replication, and sample sizes. The conclusions must be drawn appropriately based on the data presented.*

*Reviewer #1: No*

*Reviewer #2: Partly*

R1: A minimum of 10 sequences of each marker from each study site was used in the analysis. Previous published work on population genetic structure analysis have used sample numbers between 6-10 from a single study location and have obtained successful results. I suggest changing the following sentence in the discussion and conclusion in line 342-343, 370-373, 375-377–

‘Gene flow was evident with lack of population differentiation even between geographically distant populations of *P. argentipes* perhaps due to absence of geographic barriers and the continuity of habitats.’

‘Results also validated the use of *cox 1, cytb* genes and ITS2 region as a tool in understanding the population genetic structure of Phlebotominae sand flies in Sri Lanka.’

As,

Both *cox 1* and ITS2 indicated a gene flow with lack of population differentiation even between geographically distant populations of *P. argentipes* perhaps due to absence of geographic barriers and the continuity of habitats.

Results showed the possible use of *cox 1, cytb* genes and ITS2 region as markers in determining the population genetic structure of Phlebotominae sand flies in Sri Lanka.

*Q2: Is the manuscript presented in an intelligible fashion and written in standard English?*

*Reviewer #1: Yes*

*Reviewer #2: No*

R2: The typographical and grammatical errors suggested by the reviewers were corrected and changes are highlighted in the manuscript.

*Abstract
Q3: Line 30: Two mitochondrial genes viz. Please correct this sentence, I consider the word “viz.” it is misused.*

R3: Agreed and changed the sentence in line 30.

*Q4: Line 31: please delete oxidase, just leave Cytochrome b (CytB)*

R4: Agreed and deleted it in line 31.

*Q5: Line 30-31: parentheses are not italicized*

R5: Agreed and done.

*Q6: Line 33: Change Network to network*

R6: Done it in line 33.

*Q7: Line 35: change flies to sand flies*

R7: Done it in line 35.

*Q8: Line 38-42: Please restructure the sentences, because it is a bit confusing.*
R8: Changed the sentence in line 38-42.

*Introduction
Q9: Line 51-54: I suggest change the paragraph this way:
Phlebotomus argentipes (Diptera: Psycodidae) is the known vector of Leishmania donovani, the causal agent of the clinical form VL which is considered the second parasitic disease cause of a high number of deaths, in India, Nepal and Bangladesh [4].*

R9: Agreed and done the changes to line 50-52.

*Q10: Line 70: change weren´t to were not*

R10: Agreed and done in line 65.

*Q11: Line 78, 83, 103: delete this space*

R11: Deleted spaces*.*

*Q12: Line 86: Colombia is not an Old world country, please make the correction. Additionally there are more barcode studies for species from the new world, such as USA, Mexico, Brazil, please keep it in mind.*

Q12: Done the changes to the sentence in line 75-77.

*Q13: Line 99: change phylogeograpy to phylogeography*

R13: Changed as ‘phylogeography’ in line 85.

*Q14: Line 61-77: Although the information is relevant is very long, please be more specific.*

R14: Agreed and done in line 59-70.

*Q15: Line 93-102: I suggest that only provide the information regarding the relevance of the use of the cox1, cytb and ITS2 genes for the genetic characterization of Phlebotomus argentipes. And do not include information that is not relevant in your research. For example: Line 94-96:In South America the phylogenetic analysis of cox 1 sequences of Verrucarum species of New World sand flies of the genus Lutzomyia was investigated [20].Line:97-98: Mitochondrial introgression in the Lutzomyia townsendi in Colombia was studied using cytb gene [12].*

R15: Due to restricted studies in *P. argentipes* corrected statement in line 83-85.

*Q16: Line 48-107: In general, the introduction is very long, and contains information that is not very relevance. I kindly suggest that it be summarized a bit to make it more clear and precise.*

R16: The changes were made according to the comments suggested by the reviewers to the introduction to be summarized.

*Q17: Line106-107: “to study the effect of evolutionary forces that may spread resistance genes from one population to another”.
I have a question. The genes to be amplified are not necessarily genes that are related to insecticide resistance. How do you plan to evaluate and/or justify that the genetic variability that you may find are related to resistance to insecticides?*

R17: There is no link between the genes used in the study and the insecticide resistance genes. This statement was made as there is a high chance of entering such genes from one populations to another population in the absence of population genetic structure variation.

*Q18: It is not very clear to me. Please in the part of your introduction, where you talk about insecticides could explain what genes are useful for do this objective.*

R18: Explained in line 67-69. *Material and Method
Q19: Line 111-116: A total of 126 of adult sand flies were collected, this sentences is a result not is part of the method. Please move this information to the next section. Also specify how many days you collected in each of those locations.*
R19: Moved it to the result section and added information to the material and method in line 92-97.

*Results
Q20: Line:189-190: A total of 126 of adult sand flies were collected and were identified as Phlebotomus argentipes [15, 25, 26].*

R20: Added it to line 171.

*Q21: Please, also specify how many specimens you collected in each one of the four locations that you studied. Why you did not collect other sand fly species and only obtain specimens of Phlebotomus argentipes, this is very common in other studies? There are not other sand fly species in your country? How many males and females did you collect? Did you analysis 126 specimens by PCR or how many?*

R21: About 42 specimens were collected in each one of the four locations with a total of 52 females and 74 males. There are *Sergentomyia spp*. in the country. However, trapping method was basically effective for cattle-baited net trap. Thus, *Phlebotomus argetipes* were found apart from one or two *Sergentomyia spp.* specimens collected from light traps in some days, not routinely. Due to absence of sufficient numbers of samples, *Sergentomyia spp*. was not included in the study. Due to the study were based on the vector species, *Phlebotomus argentipes*, a total of 126 species were analysed by PCR followed by the analysis and interpretation were based on vector control strategies.

*Q22: Line193: how did you select those 10 specimens of the best quality?*

R22: The chromatograms of each and every sequence was checked manually and only the good quality sequences were used in the study.

*Q23: Line 214, 217,305,306,308: Change flies or fly to sand flies or sand fly according to the : case in the entire document.*

R23: Agreed and corrected.

*Q24: Line 217: correct P. argentipes*

R24: Corrected it line 199.

*Q25: Line 237: In the table, please include the number of specimens that you analyzed, for each gene and locations.*

R25: Added to the Table 1.

*Discussion
Q26: Line 322: two clades*

R26: Agreed and done it in line 302.

*Q27: Line 325: network analysis*

R27: Done it in line 305.

*Q28: Line 326: P. argentipes were closely related to specimens of the same species collected in Sri Lanka, India and Israel.*

R28: Corrected it in line 306-307.

*Q29: Line 327: of three sand flies*

R29: Corrected it in line 307.

*Q30: Line 327-328: cytb is a marker that shows variability at the intraespecific level, different from cox1 that shows differences at the interespecific level. So, why is relevant that some specimens are similar to specimens from Madagascar? Explain more about it, although you have a small sample, it is interesting that you have haplotypes similar to a locality that is considerably distant from your collection point.*

R30: The larger sample will be used for further studies to confirm this specimens exactly from Madagascar or not. If so, there is a genetic exchange may apply in between those specimens.

*Q31: Line 329: did not*

R31: Corrected it in line 309.

*Q32: Line 330: Add a sentence highlighting that your work is one of the first sequences for this gene and for this species. I think it is very important that you highlight this contribution of your work.*

R32: Done it in line 311-312.

*Q33: Line 345: does not.*

R33: Corrected it in line 327.

*Q34: Line 352-353: put the name of the area of study not numbers, because you did not listed before.*

R34: Agreed it and corrected in line 334-335.

*Q35: Line 354-360: Although it is an interesting hypothesis, I think it is very risky to assert something of this style, given that the number of specimens that you analyzed is very small. I do not consider that ten specimens are enough to think that there is gene flow in all communities of sand flies and those possible resistance genes are being inherited. Additionally, you did not analyze genetic markers for resistance.*

R35: Previously published work on population genetic structure analysis have used sample numbers between 6-10 from a single study location and have obtained successful results. This study used a minimum of 10 sequences from each marker from each locality. However, as the sample size is not very high it was stated as “possible” gene flow.

Insecticide resistance genes was taken as an example gene that could be introduced into other populations due to gene flow.

*The sentence, “*The possible gene flow revealed in the current study demonstrate the risk of dispersal of such resistance genes between populations with the potential for the spread of insecticide resistance and implications for future vector control” could be changed as *“*The possible gene flow revealed in the current study demonstrate the risk of dispersal of genes such as insecticide resistance genes between populations with the potential for the spread of insecticide resistance and implications for future vector control” in line 339-342 in the discussion.

*Q36: Line 363-373: This information is not informative for this article, I suggest that you focus on the group of sand flies and compare your results with those of other researchers. I suggest you read the article by:
1. Pech-May et al. 2016. Genetic structure and divergence in populations of Lutzomyia cruciata, a phlebotomine sand fly (Diptera: Psychodidae) vector of Leishmania mexicana in southeastern Mexico* [*https://doi.org/10.1016/j.meegid.2013.02.004*](https://doi.org/10.1016/j.meegid.2013.02.004) *2. Wolkoff, 2018. Population structure, demographic history, and environmental niche of the sand fly disease vector Lutzomyia shannoni (Dyar) (Diptera: Psychodidae) in the U.S., Mexico, and Colombia* [*https://scholarworks.uttyler.edu/biology_grad/53/*](https://scholarworks.uttyler.edu/biology_grad/53/)

R36: Added information to line 351-358.

*Q37: Line 362-382: I suggest that you change these paragraphs as follows and fill them in with additional information, so that you can keep comparing your results.
The molecular markers have been extensively used for genotyping and study of evolutionary history of mosquitoes [30, 31]. The analysis using cytb gene has demonstrated been useful to detect the lack of population differentiation and the presence of gene flow in some species of dipterans in South-east Asia [32]. The gene flow between species of insect vectors may lead to alterations in the disease patterns [35]. Low genetic structure variation in some sand fly species as a result of geographical isolation and restricted gene flow may have led to reduced flight abilities and formation of cryptic species that ultimately influence the capacity to transmit parasites such as Leishmania in Latin America [36].*
R37: Added information to line 344-351.

*Q38: Line 384-391: again, I do not agree with these statements, I suggest that you approach it in another way for greater support, or that you only suggest it as a possible hypothesis in a more subtle way.*
R38: Corrected it in line 360-366.

*References
Q39: Please check the structure of the citations again, to make it homogeneous. Scientific and gene names are also missing in italics, and there are spelling mistakes (e.g. Line 4,5,12, 16, 17, 26, 27, 38, 39)*

R39: Agreed and done it.
