# Supplementary material for: Genetic diversity and population structure of Phlebotomus argentipes: Vector of Leishmania donovani in Sri Lanka
Source: PLoS One. 2021 Sep 16;16(9):e0256819. doi: 10.1371/journal.pone.0256819 (PMC8445420; doi:10.1371/journal.pone.0256819)
Supplement: Attachment — Submitted filename: Response to Reviewers.docx [file pone.0256819.s006.docx]

***Author responses to journal requirements and additional editor’s comments:***

*Journal Requirements:*

*Q: Please review your reference list to ensure that it is complete and correct. If you have cited papers that have been retracted, please include the rationale for doing so in the manuscript text, or remove these references and replace them with relevant current references. Any changes to the reference list should be mentioned in the rebuttal letter that accompanies your revised manuscript. If you need to cite a retracted article, indicate the article’s retracted status in the References list and also include a citation and full reference for the retraction notice.*

R: Reviewed the reference list and it was completed and corrected. Following changes were made in the reference list.

- Journal name abbreviation was corrected in line 428, 443.
- Page numbers were added in line 467.
- Journal name and page numbers were added in line 473.
- Journal name, issue, page numbers and DOI number were added in line 475-476.

Following reference was removed from the reference list as it contains unpublished work. Thus, the statement in line 353-358 referred to as “unpublished work” in line 358.

Wolkoff. Population structure, demographic history, and environmental niche of the sand fly disease vector *Lutzomyia shannoni* (Dyar) (Diptera: Psychodidae) in the U.S., Mexico, and Colombia. University of Texas at Tyler; 2018.

*Additional Editor’s Comments:*

*Q1: I agree with the authors on that the target genes analyzed are good for the specimen’s identification and for the analyses of the genetic divergence within and among phlebotomine sand fly populations, but not on the gene flow. Indeed, Weeraratne and colleagues only hypothesized the presence of the gene flow using the cox1, whilst Manni et al., define that SSR markers are, as expected, more informative than ITS2 in revealing the slight genetic diversity between native and derived populations both in terms of variability and differentiation. The authors have to be more cautious when talk on the gene flow. I suggest, for example, putting your considerations as a hypothesis of the “existence of a potential gene flow” based on the results obtained of the genes analyzed, and that the gene flow have to be further investigated or supported by the analysis of microsatellites.*

*See also the article Prudhomme et al., “Altitude and hillside orientation shapes the population structure of the Leishmania infantum vector Phlebotomus ariasi.” Scientific Reports, 2020 DOI: 10.1038/s41598-020-71319-w.*

R1: Authors agreed and suggested changes have been added in the abstract section in line 32-42 and in the conclusion section in line 369-373 and 374-385.

“Therefore, there is a high risk of transferring genes of functional significance, such as those that confer insecticide resistance from one population to another with the risk of eventually spreading across the country. However, confirmation of such an assumption would require further investigations and supportive evidence probably with the use of more neutral genotyping markers such as microsatellites, which have been used in other *Phlebotomus* sp. for similar investigations including for those related to the influence of geographic distance and altitude (Prudhomme *et al.,* 2020*)*. The study also confirms the possible use of *cox 1, cytb* genes and ITS2 region as genotyping markers for the determination of the population genetic structure of Phlebotominae sand flies in Sri Lanka. Thus, the findings will pave the way for more extensive investigations, probably with the use of more genotyping markers, to aid a vector control strategy that may need to be adopted within a future national leishmaniasis control programme.”

*Q2: Change the title: since sand flies are vector of pathogen and not of diseases*

R2: The title was changed into ‘Genetic diversity and population structure of *Phlebotomus argentipes:* vector of *Leishmania donovani* in Sri Lanka.’

*Q3. Lines 36-37: I suggest changing “revealed gene flow” into “may indicate a potential gene flow”, due to the motivation above, and throughout the ms.*

R3: Changed as ‘may indicate a possible gene flow’ in line 37, 39-40, 333-334, 337, 339-343, 363-364, 372.

**Authors suggested to acknowledge funding source in the Acknowledgements section in line 391-394.**
